# S-antigen specific T helper type 1 response is present in Behcet’s disease

**Published:** 2008-08-07

**Authors:** Changlin Zhao, Peizeng Yang, Hao He, Xiaomin Lin, Bing Li, Hongyan Zhou, Xiangkun Huang, Aize Kijlstra

**Affiliations:** 1 Zhongshan Ophthalmic Center, Sun Yat-Sen University, Guangzhou, P. R. China; 2 The First Affiliated Hospital, Chongqing Medical University, Chongqing, P. R. China; 3 Eye Research Institute Maastricht, Department of Ophthalmology, University Hospital Maastricht, Maastricht, The Netherlands; 4 Animal Sciences Group, Wageningen UR, The Netherlands

## Abstract

**Purpose:**

To investigate the frequency and phenotypic and functional characteristics of S-antigen (S-Ag) specific T cells in patients with Behcet’s disease (BD).

**Methods:**

Blood was taken from 23 active BD patients, 12 inactive BD patients, and 14 healthy controls. The clinical features of the patients were summarized. T cell response against 40 mixed S-Ag peptides was identified by interferon gamma (IFN-γ) enzyme-linked immunospot assay (ELISPOT). CD69 and CD45RO were used to characterize the phenotype of S-Ag specific T cells. The functional property of S-Ag specific T cells was investigated by measuring the production of cytokines.

**Results:**

Response to the mixed S-Ag peptides was found in 56.5% and 25% of active and inactive BD patients, respectively. The responsiveness to S-Ag peptides was unrelated to the clinical features of the patients. About 65.8% of IFN-γ^+^ CD4^+^ T cells in active BD patients expressed CD69 and CD45RO concomitantly. S-Ag peptides significantly induced a production of IFN-γ and tumor necrosis factor (TNF)-α but not interleukin (IL)-2, IL-4, and IL-17 by peripheral blood mononuclear cells (PBMCs) in active BD patients with a response to S-Ag.

**Conclusions:**

S-Ag specific T cells are present in certain active BD patients, and most of them are activated memory CD4^+^ T cells. These T cells may be involved in the pathogenesis of BD via producing Th1-dominant cytokines.

## Introduction

Behcet’s disease (BD) is a chronic, relapsing, multisystem inflammatory disorder characterized by recurrent episodes of severe intraocular inflammation, oral ulcers, genital ulcers, and skin lesions. Extensive studies have provided accumulating evidence to support the notion that autoimmune response plays a major role in its pathogenesis [1-3]. However, studies on autoantigen-induced cellular immune responses in patients with BD have mainly used assays that measure the lymphoproliferative response. Arrestin, a 48 kDa soluble retinal antigen (S-Ag), is an extremely potent uveitogenic antigen among various candidate antoantigens in BD [4]. Lymphocyte proliferative responses to S-Ag or peptides derived from it have been reported to be present in a variety of human uveitis entities, including BD [5-8]. Nevertheless, the autoantigens used in most of these studies are homologous bovine proteins because it is difficult to obtain sufficient amounts of the native human protein. It is uncertain whether the epitopes from bovine S-Ag are identical with those from the human protein [5]. Studies by Doekes et al. [9], for instance, showed that sera from human uveitis patients showed preferential reactivity to human S-Ag while being very weakly reactive with bovine S-Ag. Additionally, the functional features such as cytokine secretion of autoreactive T cells against human S-Ag in human uveitis have not been investigated.

In this study, we synthesized human S-Ag overlapping peptides as the antigen and adopted enzyme-linked immunospot assay (ELISPOT), intracellular cytokine staining (ICS), and enzyme-linked immunosorbent assay (ELISA) techniques to investigate the frequency, phenotype, and functional feature of S-Ag specific T cells in BD patients.

## Methods

### Subjects

Thirty-five male BD patients with an average age of 31 years were recruited from the Uveitis Study Center of Sun Yat-sen University (Guangzhou, Guangdong, China). Fourteen healthy male individuals with an average age of 33 years were used as the controls. All BD patients met the diagnostic criteria established by the International Behcet’s Disease Study Group [10]. Twenty-three patients treated irregularly before being referred to the Uveitis Study Center showed active recurrent uveitis as evidenced by dust keratic precipitates (100%), flare and cells in the anterior chamber (100%), hypopyon (17.4%), vitreous cells (47.8%), and retinal vasculitis observed clinically or disclosed by fluorescein angiography (100%). The exact treatment history of these patients could not be retrieved. Besides recurrent oral ulceration, these active BD patients had at least one of the following extraocular manifestations: multiform skin lesions (69.6%), recurrent genital ulceration (34.8%), arthritis (21.7%), and a positive pathergy test (17.4%). They were treated with low dose prednisone (20 mg/day or less) or without systemic treatment at the time of blood sampling. Twelve patients who showed neither active intraocular inflammation nor extraocular findings for at least three months after treatment with immunosuppressive agents (prednisone 5–20 mg/day and cyclosporine A 2.0–4.0 mg/kg/day or in combination with one of the following medicines including cyclophosphamide 50 mg/day, chlorambucil 2 mg/day, and colchicine 0.5 mg/day) at the Uveitis Study Center were defined as inactive BD patients. These patients had also suffered panuveitis (100%), hypopyon (25%), recurrent oral ulceration (100%), multiform skin lesions (66.7%), recurrent genital ulceration (58.3%), arthritis (25%), a positive pathergy test (41.7%), and epididymitis (16.7%) before our treatment. Sampling was performed from the BD patients and healthy controls after they had given their informed, written consent. All procedures were approved by the ethics committee of the Zhongshan Ophthalmic Center (Guangzhou, Guangdong, China).

### Peptides synthesis and preparation

Forty overlapping peptides spanning the entire length of human retinal S-Ag sequence (P10523)  were synthesized by Shanghai Sangon Biologic Engineering Technology & Services Company (Shanghai, China). Each peptide determinant measured 20 amino acids (aa) in length and overlapped the previous sequence by 10 aa except for the last one that measured 15 aa. These peptides were synthesized by conventional solid-phase chemistry and purified by reverse-phase high-performance liquid chromatography to at least 95% purity. The endotoxin levels of the forty peptides were less than 0.001 ng/μg as determined by the Limulus amebocyte lysate assay.

A series of concentrations including 50, 20, 10, 5, and 1 μg/ml of peptides were used in the ELISPOT assay in preliminary experiments. The results showed that an optimal concentration was obtained at a concentration of 5 μg/ml, and we therefore used this concentration in the following experiments.

### Isolation of peripheral blood mononuclear cells

Peripheral venous blood was drawn in a heparinized tube from all subjects. Peripheral blood mononuclear cells (PBMCs) were isolated immediately by Ficoll–Hypaque density gradient centrifugation (Pharmacia Biotech, Shanghai, China) and suspended at 2×10^6^ cells/ml in complete culture medium (RPMI 1640 containing 10% fetal calf serum, 100 U/ml penicillin, and 100 mg/ml streptomycin).

### Interferon (IFN)-γ ELISPOT assay

The interferon (IFN)-γ ELISPOT assay was performed using a commercially available kit (BD PharMingen, San Diego, CA) according to the manufacturer’s instructions. In this study, a total of 2×10^5^ fresh PBMCs were added to microwells in triplicate and incubated with S-Ag peptides plus 1 μg/ml anti-CD28 monoclonal antibody (mAb; Clone CD28.2, BD PharMingen) for 20 h. PBMCs stimulated with 10 ng/ml anti-CD3 (Clone HIT3a, BD PharMingen) plus 1 μg/ml anti-CD28 mAbs served as positive controls. Results of preliminary experiments showed that there was no difference in IFN-γ production by PBMCs stimulated with or without 1 μg/ml anti-CD28 mAb. In further experiments for this study, PBMCs cultured alone were therefore used as negative controls.

The spots were counted automatically using an ImmunoSpot Image Analyzer (Cellular Technology Limited, Cleveland, OH). Digitized images were analyzed for the presence of areas in which color density exceeds background by an amount set on the basis of the comparison of experimental wells and negative control wells. A spot called spot-forming cell (SFC) represented an IFN-γ producing cell. The number of spots in negative control wells was at a range of no spots to two spots. Wells containing more than two spots were considered as showing a positive response.

### Cytokines assay by ELISA

PBMCs were stimulated with the mixed S-Ag peptides plus 1 μg/ml anti-CD28 mAb for 72 h at a density of 2×10^6^ cells/ml. The PBMCs, which were not stimulated, served as negative controls. The levels of IFN-γ, interleukin (IL)-2, TNF-α, IL-4, and IL-17 in cell culture supernatants were detected using the Duoset ELISA Development Kit (R&D system, Minneapolis, MN) according to the manufacturer's instruction. The lower detection limit for the IFN-γ, IL-2, TNF-α, and IL-17 assay kit was 15.6 pg/ml and for IL-4 was 31.2 pg/ml.

### Intracellular cytokine staining and flow cytometry analysis

In vitro PBMC stimulation with antigen and intracellular cytokine staining were generally performed according to the method described by Waldrop et al. [11]. Since S-Ag specific T cells were not detectable after 6 h incubation, a longer incubation time (20 h) was employed for this experiment [12]. PBMCs were stimulated with peptides plus anti-CD28 mAb as described above. The unstimulated PBMCs were used as negative controls. After a 20 h stimulation, 3 μg/ml brefeldin A (eBioscience, San Diego, CA) was added for an additional 4 h incubation to inhibit protein transport of cytokines. The PBMCs were incubated with PerCP-conjugated anti-CD3 (BD PharMingen), PeCy7-conjugated anti-CD8 (eBioscience), FITC-conjugated anti-CD69 (eBioscience) and allophycocyanin (APC)-conjugated anti-CD45RO (eBioscience) for 30 min in the dark at 4 °C. For intracellular IFN-γ detection, the cells were then fixed with 4% paraformaldehyde for 8 min at room temperature and permeabilized with 0.1% saponin (permeabilization buffer, eBioscience) overnight at 4 °C and stained with PE-conjugated anti-IFN-γ (eBioscience) or matched isotype control mAb (eBioscience) for 30 min at 4 °C. The samples were subjected to flow cytometry, and 600,000 events were collected for analysis using CellQuest 4.3 software (Becton Dickinson, San Jose, CA).

### Statistical analysis

Data were analyzed using statistical software SPSS 10.0 (SPSS Inc., Chicago, IL). Data are expressed as mean±SD. Statistical analysis was performed using Student’s *t*-test, one-way ANOVA, and Pearson correlation assay. A level of p<0.05 was considered significant.

## Results

### Specific T cell immune response to S-Ag in Behcet’s disease patients

The first set of experiments was done to identify the specific T cell response to human S-Ag in BD patients and healthy controls using the IFN-γ ELISPOT assay. PBMCs were stimulated with S-Ag synthetic peptides plus anti-CD28 mAb. In terms of the IFN-γ SFC, 56.5% (13/23) of active BD patients and 25% (3/12) of inactive BD patients showed a response to these peptides with a mean SFC frequency of 12.6/2×10^5^ (SD=5.0 / 2×10^5^) and 11.3/2×10^5^ (SD=4.2 / 2×10^5^), respectively. No healthy controls showed a positive response to these peptides (SFC 0.2±0.4/2×10^5^). The frequency of IFN-γ producing cells in active BD patients was similar to that observed in the inactive individuals. The responsiveness of PBMCs to S-Ag peptides seemed not to be affected by ocular involvement or systemic involvement in these patients (Table 1).

**Table 1:** Clinical features of patients with Behcet’s disease and the responsiveness of peripheral blood mononuclear cells against S-Ag peptides stimulation.

**Patient number**	**Age (years)**	**Diagnosis**	**Special ocular manifestations**	**Extraocular symptoms**	**Response to S-Ag**
1	35	active BD		*, **	Positive
2	30	active BD	vitreous cells	*, **	Positive
3	20	active BD	vitreous cells	*, **, ***, ##	Positive
4	17	active BD	hypopyon, vitreous cells	*, ***, #	Positive
5	25	active BD	vitreous cells	*, **	Positive
6	16	active BD	vitreous cells	*, **	Positive
7	27	active BD	hypopyon, vitreous cells	*, **	Positive
8	24	active BD	vitreous cells	*, ***, #	Positive
9	22	active BD		*, **	Positive
10	29	active BD		*, **	Positive
11	36	active BD		*, ##	Positive
12	44	active BD		*, ***	Positive
13	26	active BD		*, **, ##	Positive
14	17	active BD	vitreous cells	*, **, ***	Negative
15	40	active BD	hypopyon, vitreous cells	*, **, ***, ##	Negative
16	33	active BD		*, ***	Negative
17	27	active BD		*, ##	Negative
18	24	active BD		*, #	Negative
19	25	active BD	vitreous cells	*, **, #	Negative
20	19	active BD		*, **	Negative
21	36	active BD	hypopyon, vitreous cells	*, **	Negative
22	29	active BD		*, **, ***	Negative
23	28	active BD		*, **	Negative
24	42	inactive BD		*, ***, ##	Positive
25	29	inactive BD	hypopyon	*, **	Positive
26	37	inactive BD		*, **	Positive
27	41	inactive BD		*, **, ***	Negative
28	34	inactive BD		*, **, #, ##, ###	Negative
29	44	inactive BD	hypopyon	*, **, ***, #, ##	Negative
30	39	inactive BD		*, **	Negative
31	47	inactive BD		*, #	Negative
32	32	inactive BD	hypopyon	*, **, ***, #	Negative
33	34	inactive BD		*, ***	Negative
34	34	inactive BD		*, ***	Negative
35	39	inactive BD		*, **, ***, #, ###	Negative

In the “Extraocular symptoms” column, the asterisk indicates recurrent oral ulceration, the double asterisk designates multiform skin lesions, the triple asterisk indicates recurrent genital ulceration, the sharp (hash mark) shows a positive pathergy test, the double sharp designates arthritis, and the triple sharp indicates epididymitis.

**Figure 1:**
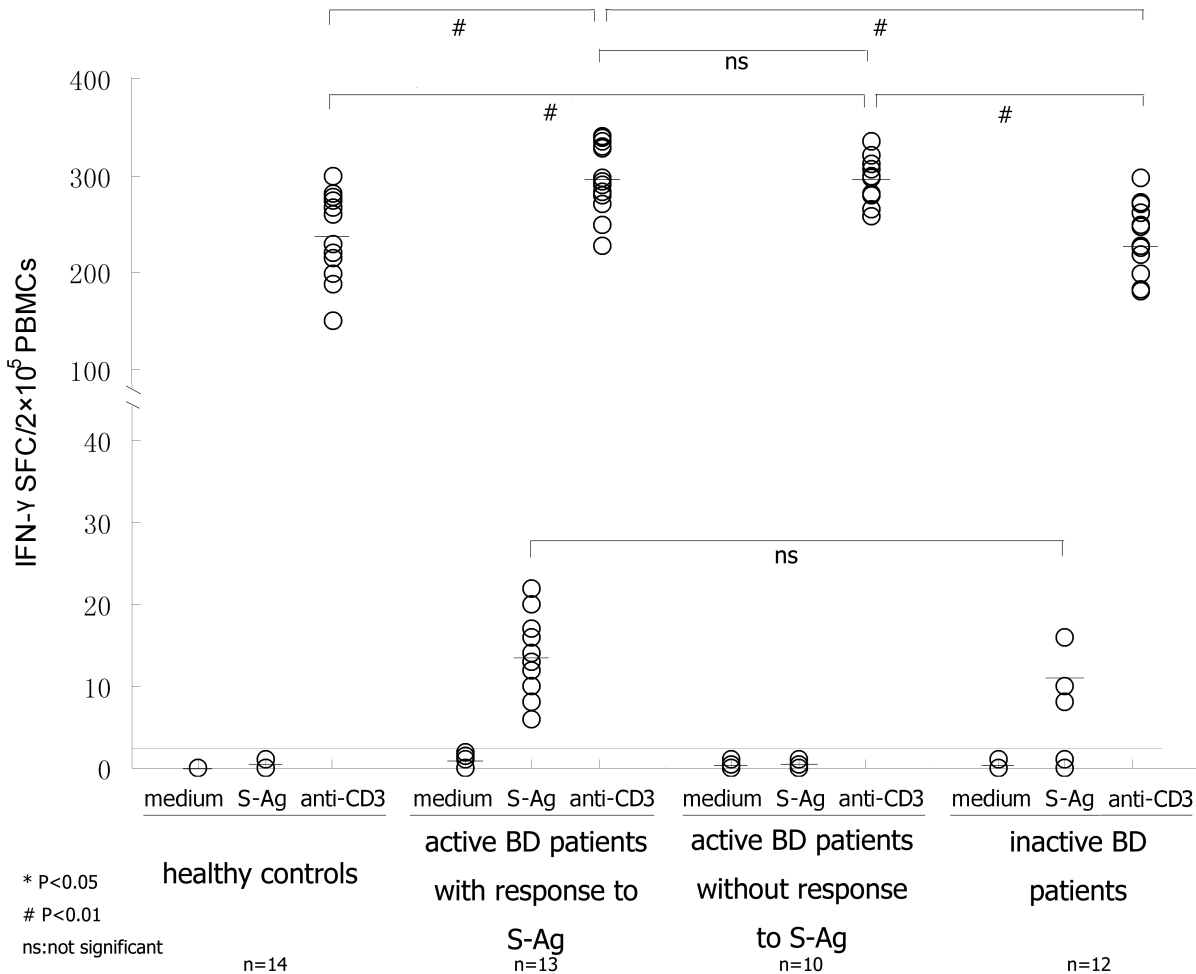
The frequency of IFN-γ producing cells detected by ELISPOT assay. Fresh PBMCs were cultured with mixed S-Ag peptides plus anti-CD28 mAb. PBMCs cultured with the medium and anti-CD3 plus anti-CD28 mAbs, respectively, were used as negative and positive controls. If the value of IFN-γ SFC was more than 2, then it was considered a positive well. Upon S-Ag peptides stimulation, 13 active BD patients and three inactive BD patients showed a positive response and the frequency of S-Ag specific T cells was similar between the two groups of patients (p>0.05, Student’s *t*-test). Upon anti-CD3 stimulation, all the subjects responded positively and the number of IFN-γ SFC was larger than that stimulated by S-Ag peptides. The frequencies of IFN-γ producing cells in four groups of PBMCs upon anti-CD3 stimulation were analyzed by ANOVA. Significant statistical difference was set at p<0.05 (*) and p<0.01 (#); ns means not significant while bars represent mean values.

To further investigate the role of S-Ag specific T cells in uveitis with BD, we divided the active BD patients into two groups according to their responsiveness to S-Ag peptides, active BD patients with a response to S-Ag (n=13) and active BD patients without a response to S-Ag (n=10). Upon the stimulation of anti-CD3 mAb, the active BD patients both with and without a response to S-Ag produced a higher frequency of IFN-γ producing cells than healthy controls (with; p<0.001, without; p=0.001) and the inactive BD patients (with; p<0.001, without; p<0.001). No difference was found between active BD patients with or without a response to S-Ag (Figure 1).

### Phenotypic characterization of S-Ag specific T cells in active Behcet’s disease patients

**Figure 2:**
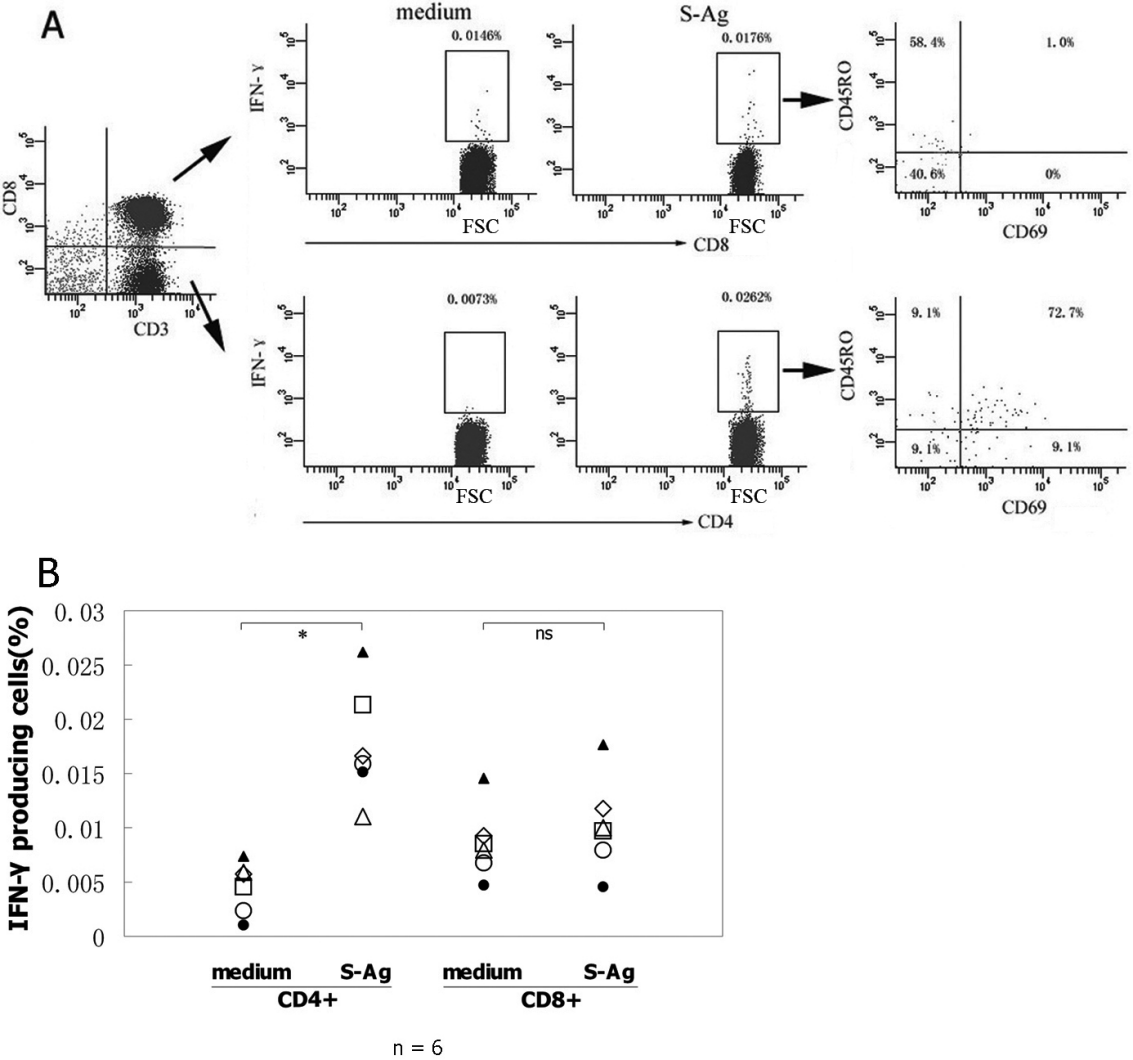
Phenotype of S-Ag specific T cells detected by intracellular cytokine staining. PBMCs from the active BD patients with a response to S-Ag were cultured with or without mixed S-Ag peptides plus anti-CD28 mAb and then were stained and examined by five color FCM. Lymphocytes were first gated using Forward Scatter (FSC) combined with Side Scatter (SSC), and then the CD3^+^CD8^-^ and CD3^+^CD8^+^ cells were gated for analyzing the expression of IFN-γ in CD4^+^ T cells and the expression of IFN-γ in CD8^+^ T cells. Lastly, the expressions of CD69 and CD45RO molecules on IFN-γ^+^CD4^+^ and IFN-γ^+^CD8^+^ T cells were analyzed, respectively. **A**: The charts show the phenotypic feature of S-Ag specific T cells of one representative active BD patient. **B**: The chart represents the expression of IFN-γ as the percentage of total CD4^+^ or CD8^+^ T cells in six different patients. Data were analyzed by Student’s *t*-test.

To determine which cell population is involved in the S-Ag specific immune response and to characterize their phenotype, PBMCs from the active BD patients with a response to S-Ag were examined by five-color flow cytometry (FCM). The expressions of CD69^+^ and CD45RO^+^ on IFN-γ producing cells were explored in CD4^+^ and CD8^+^ T cells, respectively (Figure 2A). The IFN-γ producing cells were detectable only in six of these patients by FCM. Increased IFN-γ producing CD4^+^ T cells specific to S-Ag were detected with a mean frequency of 0.0177% (range: 0.0111%–0.0262%) after stimulation with S-Ag peptides. In this subset, 83.3% (range: 76.5%–87.5%), 69.8% (range: 50%–81.8%), and 64.3% (range: 50%–75.76%) were activated T cells, memory T cells, and activated memory T cells, respectively. There was no difference in the frequency of CD8^+^ IFN-γ^+^ T cells between stimulated and unstimulated PBMCs (Figure 2B). After stimulation, 80.8%±17.8% of CD8^+^ IFN-γ^+^ T cells were early nonactivated T cells and 37.3%±18.2% of them were memory T cells.

### Pattern of cytokine production by S-Ag specific T cells

**Figure 3:**
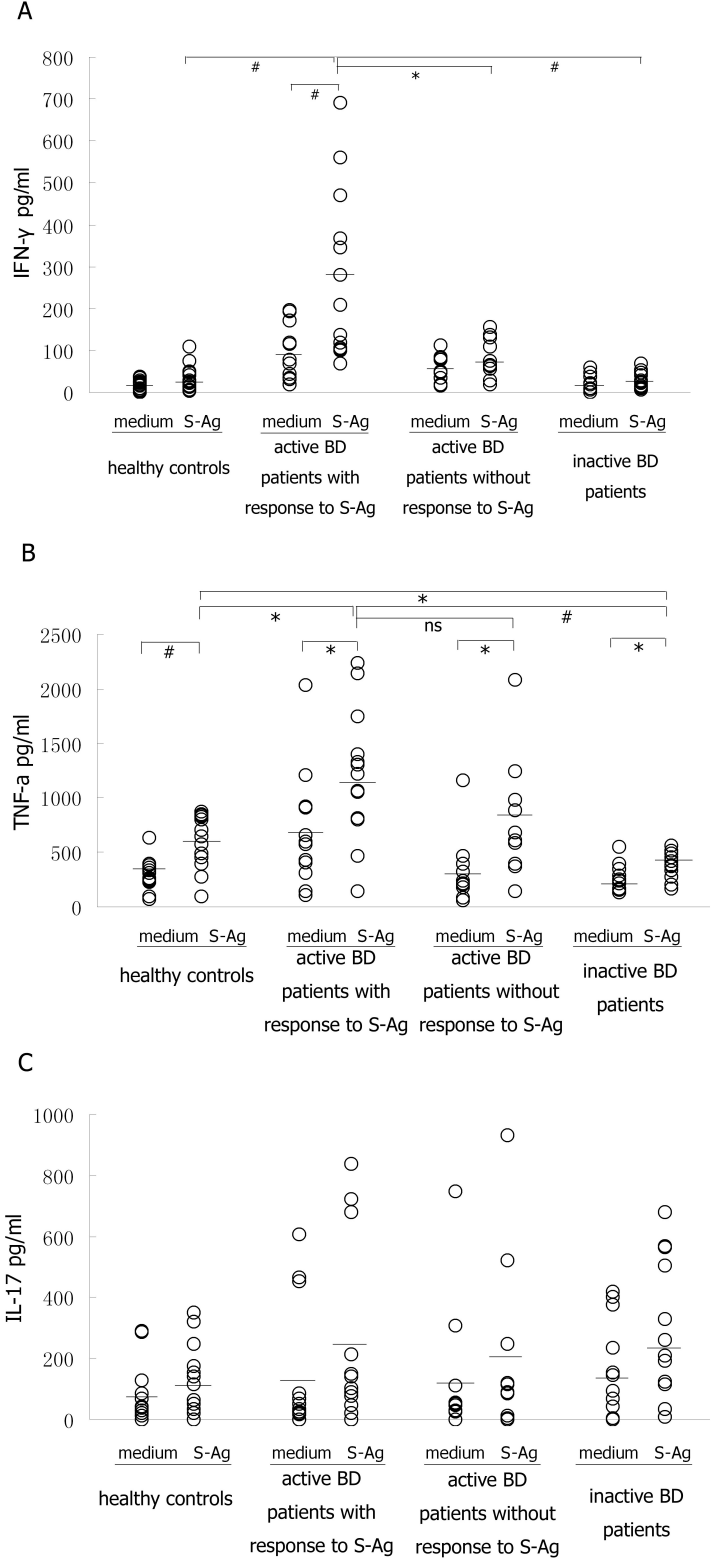
Functional property of S-Ag specific T cells disclosed on the basis of cytokine secretion. PBMCs from all subjects were stimulated with or without mixed S-Ag peptides plus anti-CD28 for 72 h. The culture supernatants were collected and assessed for the production of IFN-γ (**A**), TNF-α (**B**), and IL-17 (**C**) by ELISA. The difference in cytokine levels between the negative control and S-Ag stimulation in four groups was analyzed using Student’s *t*-test. The cytokine production by PBMCs upon S-Ag stimulation in each of the four groups was analyzed by ANOVA. Significant statistical difference was set at p<0.05 (*) and p<0.01 (#); ns means not significant while bars represent mean values.

To investigate the functional property of S-Ag specific T cells, various cytokine levels representing the function of T helper type (Th) 1/ Th2/ Th17 in culture supernatants of PBMCs from BD patients and healthy controls were measured by ELISA. The level of IFN-γ in culture supernatants of PBMCs stimulated with S-Ag peptides was significantly higher than that without stimulation in active BD patients with response to S-Ag (p=0.008; Figure 3A). Although the PBMCs produced a somewhat increased amount of IFN-γ upon the stimulation of S-Ag peptides in healthy controls, active BD patients without a response to S-Ag, and inactive BD patients, there was no difference in the IFN-γ levels between stimulated and unstimulated PBMCs from these patients. Moreover, after stimulation, the IFN-γ level in active BD patients with a response to S-Ag was higher than that in the other three groups (p=0.006, p=0.033, and p=0.005, respectively). These results confirm the S-Ag specific response at a global level and demonstrate that the mixed S-Ag peptides are immunogenic in certain BD patients.

TNF-α levels in healthy controls, active BD patients with a response to S-Ag, active BD patients without a response to S-Ag, and inactive BD patients were all elevated in response to stimulation of S-Ag peptides (p=0.001, p=0.025, p=0.033, and p=0.028, respectively; Figure 3B). Furthermore, TNF-α levels in active BD patients with a response to S-Ag was higher than that in healthy controls (p=0.025) or inactive BD patients (p=0.002). The TNF-α levels in active BD patients with a response to S-Ag was identical to that observed in active BD patients without a response to S-Ag. As for IL-17, there was a basal secretion by PBMCs from both BD patients and healthy controls (Figure 3C). Upon stimulation with S-Ag peptides, the IL-17 level was not significantly elevated in any of the subjects and the level was similar in active BD patients with and without a response to S-Ag. IL-2 and IL-4 were undetectable in all subjects.

In view of the fact that IFN-γ, TNF-α, and IL-17 can be secreted by PBMCs stimulated with S-Ag peptides, we analyzed the relationships between IFN-γ and TNF-α or IL-17 upon S-Ag stimulation in healthy controls, active BD patients with a response to S-Ag, active BD patients without a response to S-Ag, and inactive BD patients were analyzed, respectively. An analysis of Pearson correlation showed that there was no statistical correlation between IFN-γ and TNF-α or between IFN-γ and IL-17. However, certain tendencies were observed. In active BD patients with a response to S-Ag, the trend showed that IFN-γ was positively correlated with TNF-α and negatively correlated with IL-17 while in active BD patients without a response to S-Ag, the postive correlative trends were observed between IFN-γ and TNF-α or between IFN-γ and TNF-α.

## Discussion

This pilot study investigated the cellular immune response to human S-Ag at both the single cell level and the global level in BD patients with or without active uveitis. As BD is male predominant and the male patients often have severe uveitis compared with the female patients [13,14], we only selected male BD patients as the subjects to avoid the interference of hormones in the study. Since the acknowledged immunodominant epitope of human S-Ag is uncertain, we used a panel of overlapping peptides as the antigen to identify specific immune response in these patients. We found that S-Ag specific T cells existed in most active BD patients and a few of the inactive patients. Most autoreactive T cells to S-Ag were activated memory CD4^+^ T cells. The study on the functional properties revealed that S-Ag peptides induced a production of IFN-γ and TNF-α but not IL-17 in active BD patients with a response to S-Ag.

Cellular proliferative response to S-Ag or S-Ag peptides have been described in BD patients [6,8] whereas the autoimmunity against S-Ag based on cytokine-secreting has not been reported in patients with uveitis. The ELISPOT assay has proven to be an optimal technique for assessing antigen specific cells at a single cell level, which could display one target cell among one million cells [15,16]. Since IFN-γ was the key cytokine in S-Ag mediated uveitis [12,17], we applied the IFN-γ ELISPOT assay in this study to measure the cellular immune response to S-Ag in BD patients and to enumerate the frequency of S-Ag specific T cells. We further quantitated the reactivity by ELISA at the global level. Our data showed that more than one-half of the active BD patients and a quarter of the inactive BD patients responded to the mixed S-Ag peptides at a low frequency of autoreactive T cells. This response was observed in less than 30% of active Vogt-Koyanagi-Harada syndrome patients (data not shown) and none of the healthy controls. The proportion of the positive response to S-Ag is in agreement with the results presented by Yamamoto et al. [6]. They found that 61% of BD patients with uveitis and 29% of BD patients without uveitis responded to bovine S-Ag and 30% of healthy controls displayed a weakly positive reactivity. The negative responses shown by ELISPOT assay in all healthy controls may justify that it is more specific than the proliferation assay. Interestingly, although we found a similar frequency of S-Ag specific T cells in inactive and active BD patients, the IFN-γ level measured by ELISA in inactive patients was significantly lower than that observed in active patients. ELISPOT detects the cells as soon as they begin to secrete the specific cytokine upon antigen stimulation while ELISA measures the cytokine concentration in the cell culture supernatant, which sometimes has a bias due to minimal synthesis and/or cytokine consumption [18]. The S-Ag specific T cells in inactive patients may produce minimal IFN-γ at the global level, which is attributed to their functional inhibition by immunosuppressive agents. Therefore, the IFN-γ level was discrepant between ELISA and ELISPOT measurement in these patients. With regard to the anti-CD3 stimulation, active BD patients produced a higher frequency of IFN-γ producing cells than healthy controls and inactive BD patients, which may show that active BD patients were in an enhanced immune state. The result that the number of IFN-γ producing cells in active BD patients was equal whether they responded to S-Ag peptides or not may suggest that varied mechanisms besides S-Ag-mediated autoimmunity are involved in the pathogenesis of BD.

ICS is another ideal approach for assessing single cellular responses and has been used earlier to study the frequency and function of effector T cells in BD patients [12,19,20]. Following confirmation of S-Ag specific T cells by ELISPOT in active BD patients, we analyzed their phenotypic feature using multiparameter FCM for the expression of CD69 (an early activation marker) and CD45RO (a memory marker) in active BD patients with a response to S-Ag. The results showed that memory CD4^+^ T cells were the source of S-Ag specific T cells. Although the stimulated CD8^+^ T cells also produced a little amount of IFN-γ, most of them were CD69^-^ T cells. Therefore these cells were presumed as nonspecific effector lymphocytes [21]. It is worthy to point out that only approximately 50% of the active BD patients with a response to S-Ag showed IFN-γ producing cells detected by FCM. This may be due to a relatively lower sensitivity of FCM than ELISPOT to detect the antigen-specific cellular response. It is regrettable that the phenotype of S-Ag specific T cells in inactive BD patients could not be investigated due to the unavailability of blood samples.

An excessive Th1 response involving the pathogenesis of BD has been demonstrated, mainly by using polyclonal stimuli [20,22,23]. Recently, studies have disclosed that Th17 cells contribute to autoimmune uveitis [24,25] and experimental autoimmune uveitis (EAU) [26]. Data from our laboratory have shown that IL-23 promotes CD4^+^ T cells to produce IL-17 in BD patients [27]. A question is raised as to whether S-Ag specific T cells play a role through the Th17 response pathway. Therefore, we compared the cytokine profiles of Th1, Th2, and Th17 after stimulation with S-Ag peptides. Our results showed that the lymphocytes mainly showed a Th1 response as evidenced by a notable production of IFN-γ and TNF-α but little production of IL-17. Due to the highly heterogeneous cytokine production by individuals and limited sample size, there was no statistical correlation on cytokine yield between IFN-γ and TNF-α or between IFN-γ and IL-17. The interesting tendencies outlined offered us some helpful information. The IL-17 level was not coincident with the Th1 cytokines in active BD patients with or without a response to S-Ag. This result may suggest that the S-Ag specific Th1 and Th17 cells play different roles in Behcet’s uveitis. It may support the notion that a high level of IFN-γ may inhibit the generation of an IL-17 response [25,26]. The elevated TNF-α level in all active BD patients both with and without a response to S-Ag suggests that the TNF-α level might be an indication of disease activity. Studies with large group of patients may shed more light on this issue.

In summary, a low frequency of S-Ag specific T cells was displayed by ELISPOT assay in certain BD patients for the first time. This antigen specific response was evidenced by the presence of activated memory CD4^+^ IFN-γ^+^ T cells and a significant production of IFN-γ and TNF-α by PBMCs. These results suggest that S-Ag specific T cells may be involved in the pathogenesis of BD via producing Th1-dominant cytokines. As the information on the treatments of active patients was not exactly known, we were not able to determine whether the responsiveness to S-Ag as well as the phenotype and cytokine profiles of S-Ag specific T cells were affected by individual treatment history of each patient or not. Further studies are in progress to map the Th1 cell immunodominant epitope of S-Ag.
